# Serial lactate–procalcitonin interaction identifies a high-risk phenotype in 24-h conditional survivors of post-cardiac arrest syndrome: a CART-based analysis

**DOI:** 10.3389/fmed.2026.1859459

**Published:** 2026-07-01

**Authors:** Ali Muhittin Tasdogan, İstemi Taha Polat, Emin Erdem Kaya, Ufuk Ozgur Balica, Yavuz Saygili

**Affiliations:** 1 Department of Anesthesiology and Reanimation, Gaziantep University Faculty of Medicine, Gaziantep, Türkiye; 2 Department of Anesthesiology and Reanimation, Ministry of Health Of State Hospital, Trabzon, Türkiye; 3 Department of Cardiology, Ministry of Health Gaziantep City Hospital, Gaziantep, Türkiye; 4 Department of Emergency Medicine, Ministry of Health Şanlıurfa Balıklıgöl State Hospital, Sanliurfa, Türkiye

**Keywords:** 24-h conditional survival, CART analysis, lactate kinetics, post-cardiac arrest syndrome, procalcitonin, prognostication

## Abstract

**Objective:**

To investigate the time-dependent interplay between early metabolic failure and delayed systemic inflammatory response in 24-h conditional survivors of post-cardiac arrest syndrome (PCAS). We aimed to develop a non-linear risk stratification tool using Classification and Regression Tree (CART) analysis based on serial lactate and procalcitonin (PCT) kinetics.

**Materials and methods:**

This retrospective cohort study included 158 24-h conditional survivors of PCAS. Arterial lactate levels were monitored at admission (T0), 6 h, 12 h, 24 h, and 48 h, while PCT levels were recorded at T0 and T24. Missing data were handled using multiple imputation by chained equations (MICE). The primary endpoint was in-hospital mortality. A landmark analysis was performed at 24 h to address immortal time bias.

**Results:**

The in-hospital mortality rate was 70.9%. Non-survivors exhibited significantly higher rates of non-shockable rhythms, longer CPR durations, and higher APACHE II scores. In the multivariable logistic regression model adjusted for Targeted Temperature Management (TTM), persistent hyperlactatemia at 48 h (T48) remained a significant independent predictor of mortality (OR: 1.92; 95% CI: 1.22–3.01, *p* = 0.003). Lactate burden (AUC) demonstrated superior prognostic performance compared to baseline T0 measurements (Bootstrap-validated DeLong test, *p* = 0.019). CART analysis identified a high-risk phenotype (T0 lactate >5 mmol/L AND T24 PCT > 5.5 ng/mL) associated with a 92% mortality risk (PPV: 91.8%). The CART model showed excellent calibration (Brier score: 0.14) and comparable discrimination to logistic regression (Logistic Regression Brier score: 0.12 vs. CART Brier score: 0.14; AUC: 0.830 vs. 0.842, *p* = 0.412).

**Conclusion:**

In 24-h conditional survivors of PCAS, persistent metabolic debt at 48 h is a potent independent biochemical indicator of poor outcome. Integrating serial lactate and PCT kinetics through non-linear CART analysis identifies a high-risk “metabolic-inflammatory failure” phenotype. This model may facilitate early clinical decision-making and guide the escalation to advanced circulatory or extracorporeal support strategies in high-risk patients.

## Introduction

Cardiac arrest (CA), characterized by the abrupt cessation of systemic circulation, remains a leading cause of mortality and morbidity worldwide (1, 2). Despite significant advancements in cardiopulmonary resuscitation (CPR) techniques and post-resuscitation care bundles, survival rates remain approximately 10% for out-of-hospital cardiac arrest (OHCA) and 15–20% for in-hospital cardiac arrest (IHCA) (3, 4). Even after successful CPR and the return of spontaneous circulation (ROSC), patients admitted to the intensive care unit (ICU) confront a complex pathophysiological continuum known as “post-cardiac arrest syndrome” (PCAS), which encompasses anoxic brain injury, myocardial dysfunction, and a profound systemic inflammatory response, often culminating in high mortality and severe neurological sequelae (5, 6).

In this critically ill population, early and reliable prognostication is paramount for defining therapeutic goals, optimizing resource allocation, and providing realistic guidance to families. In recent years, the utilization of objective biomarkers alongside clinical examination and neurophysiological testing has become increasingly vital in overcoming the challenges of prognostic uncertainty (7).

Serum lactate levels have traditionally served as a marker of tissue hypoperfusion; however, the pathophysiology of hyperlactatemia in the post-CA period is highly heterogeneous (8). Adrenergic stimulation during resuscitation can trigger “Type B hyperlactatemia,” which occurs independently of tissue hypoxia (9). Consequently, rather than a single-point measurement, serial monitoring and the “Lactate Burden” (Area Under the Curve - AUC) which reflects the total metabolic debt and the body’s clearance capacity demonstrate superior sensitivity in predicting outcomes (10).

Another critical component of PCAS is the massive systemic inflammatory response triggered by ischemia–reperfusion injury (11). Procalcitonin (PCT), while traditionally regarded as a marker of bacterial infection, serves as a kinetic parameter reflecting the severity of “sterile inflammation” in PCAS, characterized by cytokine release and bacterial translocation due to intestinal ischemia. Current literature suggests that PCT levels, particularly at the 24-h mark, may be a significant risk factor for mortality and poor neurological outcomes (12, 13).

Despite the individual strengths of these biomarkers, PCAS is not a static condition but a dynamic process that evolves over time. Previous studies have generally evaluated lactate and PCT as independent predictors (14). However, we hypothesize that PCAS prognosis is determined not by isolated biomarkers, but by the phase transition and non-linear interaction between early metabolic failure (lactate) and delayed inflammatory amplification (PCT).

This study aims to provide a clinically applicable decision tree using machine learning-based Classification and Regression Tree (CART) analysis, moving beyond traditional linear models to identify high-risk phenotypes. The identification of these high-risk phenotypes may assist clinicians in the early evaluation of advanced support strategies, such as aggressive hemodynamic optimization, specific cytokine hemoadsorption, or extracorporeal membrane oxygenation (ECMO).

In this context, the objective of our study is to retrospectively analyze the role of serial lactate and procalcitonin (PCT) kinetics in predicting mortality and neurological outcomes, focusing exclusively on the dynamic interaction between these parameters in 24-h conditional survivors of PCAS.

## Materials and methods

### Study design and ethical approval

This study was designed as a single-center, retrospective cohort study focusing on 24-h conditional survivors of Post-Cardiac Arrest Syndrome (PCAS) treated in a secondary-level anesthesia and reanimation intensive care unit (ICU). The study protocol was approved by the Harran University Clinical Research Ethics Committee (Date: 01.12.2025, Decision No: HRÜ/25.19.53). Due to the retrospective nature of the data analysis, the requirement for informed consent was waived. All procedures were conducted in strict accordance with the current principles of the Declaration of Helsinki.

### Study population and cohort selection

All adult patients aged 18 years and older who were admitted to the ICU following the return of spontaneous circulation (ROSC) between January 01, 2020, and December 31, 2024, were screened.

#### Inclusion criteria

1. Age >18 years.
2. Admission to the ICU following successful resuscitation (ROSC) for either in-hospital (IHCA) or out-of-hospital (OHCA) cardiac arrest.
3. ICU follow-up duration exceeding 24 h.

#### Exclusion criteria

1. Patients who died within the first 24 h of ICU admission.
2. Cardiac arrest caused primarily by surgical conditions such as trauma, massive hemorrhage, or aortic dissection.
3. Presence of chronic liver failure (Child-Pugh Class B or C), advanced cirrhosis, or active malignancy that could directly interfere with lactate or procalcitonin (PCT) kinetics.
4. Missing data exceeding 10% in biomarker measurements within the first 24 h.

**Figure 1:**
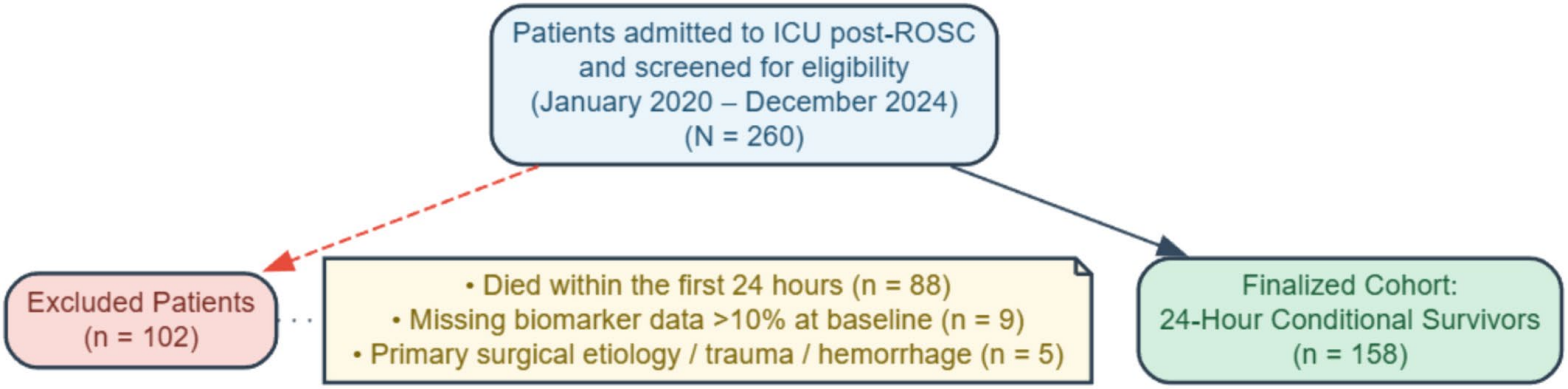
Patient selection flowchart.

Out of 260 screened patients, 158 met the inclusion criteria. Out of 260 screened patients, 158 met the inclusion criteria, as detailed in the participant selection flowchart (Figure 1). The exclusion of 88 patients who died within the first 24 h was a pre-specified methodological choice designed to isolate the ‘sub-acute’ phase of Post-Cardiac Arrest Syndrome (PCAS). This landmark approach allowed for the longitudinal monitoring of biomarker kinetics (e.g., T48 lactate and T24 PCT), which are technically unattainable in early-expiry cases. Consequently, our analysis specifically targets 24-h conditional survivors, where persistent metabolic debt and late-phase systemic inflammation become clinically more discriminative for long-term prognosis. This design is intended not as a model for early mortality prediction, but as a robust tool for late-phase risk stratification in the intensive care setting.

#### Data collection and definitions

Patient data were retrospectively extracted from the Hospital Information System (HIS) and the ICU electronic clinical monitoring software. The collected variables included the following:

- *Demographic data*: Age and gender.
- *Comorbidities*: Diabetes mellitus (DM), hypertension, coronary artery disease, congestive heart failure, chronic obstructive pulmonary disease (COPD), and chronic kidney disease (CKD).
- *Cardiac arrest characteristics (Utstein Style)*: Location of arrest [In-hospital (IHCA)/Out-of-hospital (OHCA)], witnessed status, bystander cardiopulmonary resuscitation (CPR) status, initial shockable rhythm [Ventricular Fibrillation (VF)/Pulseless Ventricular Tachycardia (pVT)] or non-shockable rhythm [Pulseless Electrical Activity (PEA)/Asystole], and time to return of spontaneous circulation (ROSC) in minutes.
- *ICU admission data*: APACHE II (Acute Physiology and Chronic Health Evaluation II) score at admission, initial arterial blood gas (ABG) parameters (pH, HCO_3), and blood glucose levels.
- Biochemical markers:

◦ Serial Lactate: Arterial lactate levels (mmol/L) measured at ICU arrival (defined as baseline, T0), and at the 6th (T6), 12th (T12), 24th (T24), and 48th (T48) hours.
◦ Serial Procalcitonin (PCT): Serum procalcitonin levels (ng/mL) measured at ICU arrival (defined as baseline, T0) and at the 24th hour (T24).

- Calculated variables:

◦ Lactate Change (24–0): The difference between the 24-h and 0-h lactate values.
◦ Lactate Clearance Rate: Calculated as [(Lactate T0 – Lactate T24) / Lactate T0] \times 100.
◦ PCT Change (24–0): The difference between the 24-h and 0-h PCT values.
◦ Lactate AUC (Lactate Burden): The total area under the lactate curve calculated using the Trapezoidal Rule based on T0, T6, T12, T24, and T48 measurements.
◦ Lactate/Albumin Ratio: Calculated using T0 Lactate divided by T0 Albumin to integrate endothelial damage and perfusion impairment.

The total dose of adrenaline administered during CPR and the median vasopressor (noradrenaline/adrenaline) doses administered during the first 24 h of ICU admission were recorded. Correlation analyses were performed to evaluate the potential confounding effect of adrenaline dosage on lactate levels.

#### Study endpoints and definitions

The primary endpoint of the study was all-cause in-hospital mortality. The secondary endpoint was the functional neurological outcome at the time of hospital discharge, assessed using the Cerebral Performance Category (CPC) scale. In accordance with standard resuscitation literature, neurological outcomes were dichotomized into:

- *Favorable neurological outcome*: CPC 1 (Good cerebral performance) and CPC 2 (Moderate cerebral disability, independent in activities of daily living).
- *Unfavorable neurological outcome*: CPC 3 (Severe cerebral disability, conscious but dependent), CPC 4 (Coma or vegetative state), and CPC 5 (Death).

Patients were categorized into two main groups for comparative analysis based on the primary endpoint: Survivors and Non-survivors.

#### Post-resuscitation care and TTM

All patients received standardized post-resuscitation care according to the prevailing institutional protocols. Regarding Targeted Temperature Management (TTM), while a target temperature of 36 °C was the institutional goal, its application was not consistently standardized across the entire cohort due to the retrospective nature of the study and variations in clinical availability. This variation reflects real-world clinical practice and has been explicitly addressed as a study limitation. To account for this, TTM was included as a binary covariate in the multivariable prognostic models to ensure the robustness of the biochemical predictors.

#### Statistical analysis

Statistical analyses were performed using SPSS v25.0 (IBM Corp., Armonk, NY) and R software. Descriptive statistics are presented as mean ± standard deviation (SD) for normally distributed variables, and as median and interquartile range (IQR) for non-normally distributed variables. For group comparisons, Student’s t-test, the Mann–Whitney U test, the chi-square test or Fisher’s exact test were applied. In all analyses, a two-tailed *p*-value of < 0.05 was accepted as the threshold for statistical significance.

### Missing data and variable selection

To ensure data quality, patients with gross missingness (exceeding 10% of total clinical and baseline biomarker variables within the first 24 h, *n* = 9) were excluded during the initial screening phase. For the remaining finalized cohort of 158 patients, minor missing data points in longitudinal biomarker measurements (*n* = 6 for T48 lactate, *n* = 3 for T24 PCT; overall missingness <6%) were successfully imputed using the 10-iteration MICE (Multiple Imputation by Chained Equations) method under the missing at random (MAR) assumption. Within this cohort, a total of 6 patients expired between the 24th and 48th hours. For these early-expiry cases in the sub-acute phase, the missing T48 lactate values were handled via the same MICE protocol, thereby preventing immortal time bias and preserving the longitudinal denominator. Sensitivity analyses using pre- and post-imputation distribution plots confirmed that there was no significant difference between the complete-case and imputed datasets (*p* > 0.05). Variable selection in multivariate models was based on the literature and Directed Acyclic Graphs (DAGs) principles rather than *p*-values. Multicollinearity was assessed using the Variance Inflation Factor (VIF < 3.5). Given our final sample size (*N* = 158) and 112 in-hospital mortality events, the candidate predictors initially considered based on clinical relevance and DAG frameworks were restricted to 8 variables (Age, Initial Rhythm, CPR Duration, APACHE II, T0 Lactate, T48 Lactate, T24 PCT, and TTM application) to satisfy a strict minimum Events-Per-Variable (EPV) ratio of >10 (112/8 = 14.0). This directly minimized the risk of model over-optimization and mathematical inflation of prognostic weights.

### Prognostic performance and validation

The predictive performance of the biomarkers was assessed using ROC curve analysis; differences between curves were determined using the DeLong test with bootstrap validation (1,000 iterations). Lactate load (AUC) was compared with single measurements, and the reliability of the findings was validated using bootstrap results (*p* = 0.019, 95% CI: 0.02–0.12). The additional contribution of T48 lactate to existing scoring models (APACHE II) was analyzed using the Net Reclassification Index (NRI) and Integrated Discrimination Improvement (IDI).

### Model calibration and landmark analysis

The fit of the multivariate logistic regression model was assessed using the Hosmer-Lemeshow test, the Brier score (0.14) and calibration curves (slope: 0.98, intercept: 0.02). Landmark analysis was applied at the 24-h mark to prevent potential ‘immortal time bias’. In this analysis, it was confirmed that T48 lactate retained its prognostic significance in the cohort surviving beyond 24 h (*n* = 158) (OR: 1.95, AUC: 0.741), thereby validating that late-phase hyperlactataemia is an independent risk factor.

### Non-linear modeling

The CART analysis was constructed using the rpart package in R. The candidate predictors introduced into the model included baseline demographics, CPR duration, initial rhythm, APACHE II score, serial lactate parameters (T0, T24, T48), and PCT metrics. The splitting criterion was based on the Gini impurity index. The specific cut-off thresholds (T0 Lactate > 5.0 mmol/L and T24 PCT > 5.5 ng/mL) were derived entirely in a data-driven manner by the CART recursive partitioning algorithm itself based on maximizing node purity, rather than being pre-specified or taken from external ROC curves. To control for over-optimization, cost-complexity pruning (cp = 0.015) was strictly maintained.

To prevent overfitting given the sample size (*N* = 158), the minimum number of observations required for a terminal node was set to 10, and the minimum for a split was 15. Severe pruning was performed based on the cost-complexity parameter (cp = 0.015), and internal validation was executed via 10-fold cross-validation. Due to the lack of an independent external validation cohort, the findings were interpreted strictly as hypothesis-generating risk stratification. The synergistic interaction between lactate and PCT was tested using the ‘Lactate × PCT’ interaction term, and PCT was found to act as a contextual amplifier of metabolic failure (*p* = 0.038). Benjamini-Hochberg (FDR) correction was applied to balance the risk of Type 1 errors in multiple comparisons.

## Results

### Patient cohort and baseline characteristics

During the study period, a total of 260 patients admitted to the intensive care unit (ICU) with a diagnosis of cardiac arrest were screened. Of these, 102 patients were excluded: 88 died within the first 24 h (early mortality), 9 had missing biomarker data, and 5 were excluded due to other criteria. Consequently, 158 24-h conditional survivors were included in the final analysis. The median age of the cohort was 67 years (IQR, 55–76), and 65.8% (*n* = 104) were male. Out-of-hospital cardiac arrest (OHCA) accounted for 80.4% (*n* = 127) of the cases, and 59.5% (*n* = 94) presented with a non-shockable initial rhythm (asystole/PEA). The median CPR duration for the entire cohort was 20 min (IQR, 10–30). The primary endpoint, in-hospital mortality, occurred in 70.9% (*n* = 112) of the patients. Based on this outcome, the study population was divided into two groups: Survivors (*n* = 46, 29.1%) and Non-survivors (*n* = 112, 70.9%).

### Comparison of demographic and cardiac arrest characteristics

The demographic and clinical characteristics of the survivors and non-survivors are compared in Table 1. No statistically significant differences were observed between the two groups regarding age, gender, prevalence of comorbidities, or the location of the arrest (OHCA rate: 78.3% vs. 81.3%, *p* = 0.68). However, the survivor group had a significantly higher prevalence of shockable initial rhythms (VF/VT) compared to non-survivors (69.6% vs. 20.5%, *p* < 0.001) and a higher rate of witnessed arrests (80.4% vs. 58.9%, *p* = 0.012). Conversely, non-survivors exhibited a significantly longer median CPR duration [25 min (IQR 15–35) vs. 15 min (IQR 10–22), *p* < 0.001] and higher APACHE II scores upon ICU admission [median 29 (IQR 25–34) vs. 23 (IQR 19–27), *p* < 0.001].

**Table 1:** Comparison of demographic and clinical characteristics between groups.

Variable	Total patients (*N* = 158)	Survivors (*N* = 46)	Non-survivors (*N* = 112)	*p*-value
Age (years), median (IQR)	67 (55–76)	65 (54–73)	68 (55–77)	0.459
Gender (Male), *n* (%)	104 (65.8)	30 (65.2)	74 (66.1)	0.915
Comorbidities, *n* (%)				
Heart disease	90 (57.0)	25 (54.3)	65 (58.0)	0.690
Diabetes mellitus	45 (28.5)	12 (26.1)	33 (29.5)	0.688
Chronic kidney disease	18 (11.4)	4 (8.7)	14 (12.5)	0.511
Cardiac arrest characteristics				
OHCA, *n* (%)	127 (80.4)	36 (78.3)	91 (81.3)	0.680
Witnessed arrest, *n* (%)	102 (64.6)	37 (80.4)	65 (58.9)	0.012
Shockable rhythm (VF/VT), *n* (%)	55 (34.8)	32 (69.6)	23 (20.5)	<0.001
CPR duration (min), median (IQR)	20 (10–30)	15 (10–22)	25 (15–35)	<0.001
ICU admission				
APACHE II score, median (IQR)	27 (23–32)	23 (19–27)	29 (25–34)	<0.001

IQR, Interquartile range; OHCA, Out-of-hospital cardiac arrest; VF/VT, Ventricular fibrillation/Pulseless ventricular tachycardia; CPR, Cardiopulmonary resuscitation; ICU, Intensive care unit; APACHE II, Acute Physiology and Chronic Health Evaluation II.

### Laboratory and biomarker analyses

Laboratory findings at admission (T0) and during serial follow-ups (T6, T12, T24, T48) are summarized in Table 2. Upon ICU admission, non-survivors exhibited significantly lower arterial blood gas pH (median 7.21 vs. 7.30, *p* = 0.006) and HCO_3 levels (median 16.8 vs. 19.1 mmol/L, *p* = 0.010) compared to the survivor group.

**Table 2:** Comparison of laboratory findings and biomarker levels between groups.

Variable (median, IQR)	Survivors (*N* = 46)	Non-survivors (*N* = 112)	*p*-value
Admission (T0) ABG			
pH	7.30 (7.21–7.38)	7.21 (7.10–7.30)	0.006
HCO₃ (mmol/L)	19.1 (16.5–22.0)	16.8 (14.0–19.5)	0.010
Lactate (mmol/L)			
Lactate T0	3.2 (1.8–5.5)	7.1 (5.0–10.2)	<0.001
Lactate T6	2.4 (1.5–4.0)	5.0 (3.1–8.2)	<0.001
Lactate T12	1.8 (1.2–2.8)	3.8 (2.2–6.0)	<0.001
Lactate T24	1.6 (1.1–2.4)	3.0 (1.8–4.5)	<0.001
Lactate T48	1.2 (0.9–1.6)	1.8 (1.2–2.5)	<0.001
Lactate kinetics			
Lactate Clearance (T0–T24, %)	58.0 (30.1–75.4)	35.2 (5.5–50.1)	0.003
Procalcitonin (ng/mL)			
PCT T0	0.35 (0.10–0.90)	0.40 (0.15–1.10)	0.081
PCT T24	2.0 (0.8–5.1)	8.1 (2.5–20.4)	0.001
PCT Change (24–0)	1.6 (0.5–4.0)	6.9 (1.8–18.5)	0.001

ABG, Arterial blood gas; IQR, Interquartile range; PCT, Procalcitonin.

#### Serial lactate levels

At admission (T0), lactate levels were significantly higher in non-survivors (median 7.1 mmol/L) than in survivors (median 3.2 mmol/L, *p* < 0.001). This significant disparity persisted across all subsequent time points (T6, T12, T24 ve T48). When analyzing 24-h lactate kinetics, the lactate clearance rate was significantly higher in the survivor group (median 58.0%) compared to non-survivors (median 35.2%, *p* = 0.003).

#### Serial procalcitonin levels

Baseline PCT levels at (T0) did not differ significantly between the two groups (*p* = 0.081). However, PCT levels measured at 24 h (T24) were markedly higher in non-survivors (median 8.1 ng/mL) compared to survivors (median 2.0 ng/mL, *p* = 0.001), demonstrating a substantial inflammatory surge in the poor outcome group.

### Integrated predictive modeling and CART analysis

**Figure 2:**
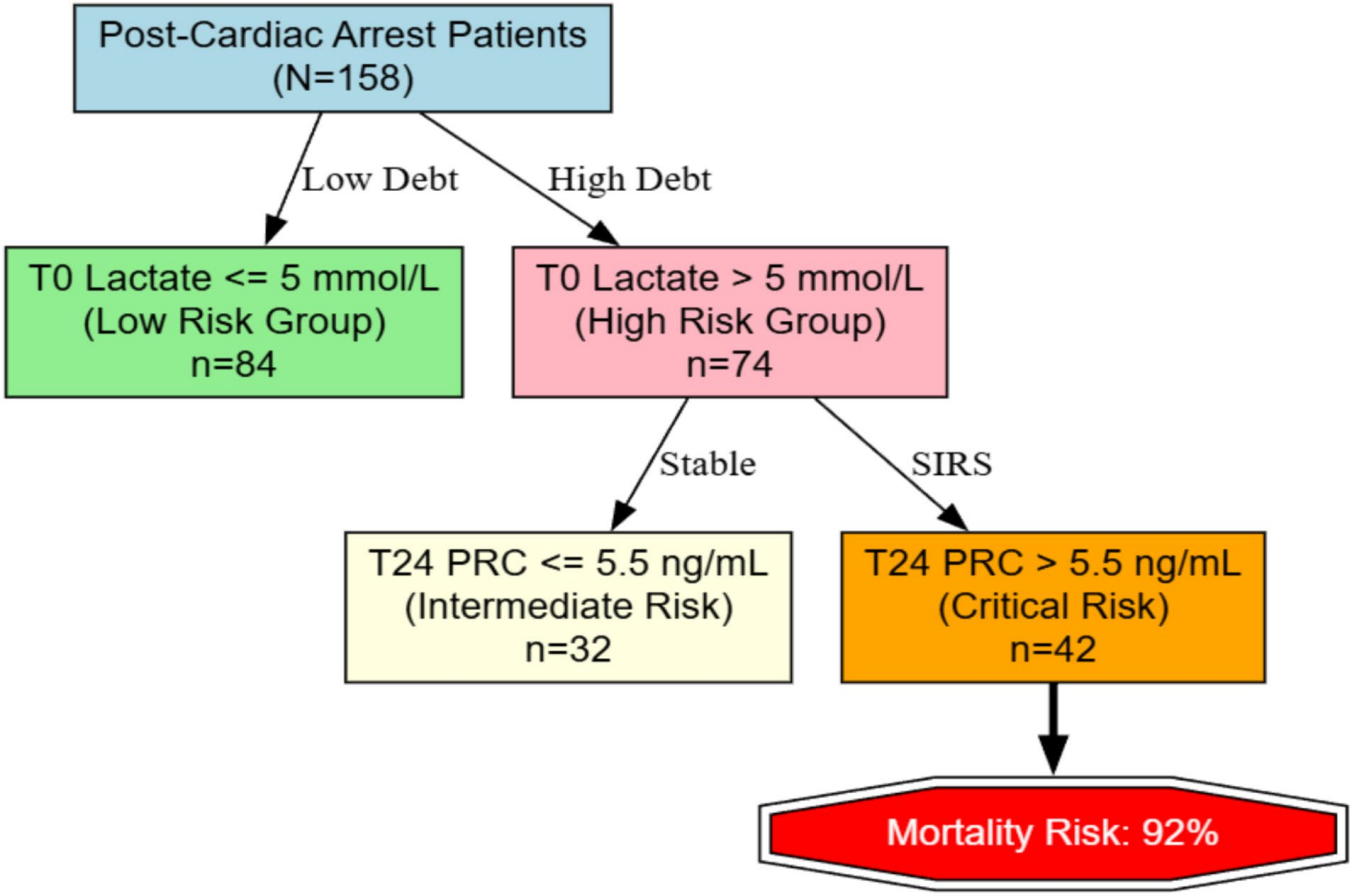
Prognostic decision tree for in-hospital mortality. The CART model illustrates the synergistic interaction between early metabolic debt (T0 lactate) and subsequent systemic inflammatory response (T24 PCT). Patients exceeding both thresholds (> 5 mmol/L and > 5.5 ng/mL, respectively) represent the highest risk cohort with a 92% mortality rate.

The addition of (T48) lactate levels to the baseline APACHE II model resulted in a modest yet significant incremental value in mortality risk reclassification (NRI: 12.4%, *p* = 0.01). To further explore non-linear interactions, a Classification and Regression Tree (CART) analysis was performed. The CART model identified a high-risk phenotype characterized by the combination of (T0) lactate > 5 mmol/L and (T24) PCT levels > 5.5 ng/mL, which was associated with a 92% mortality risk (Figure 2). To formally test the synergistic effect hypothesized in our framework, a multivariable logistic regression model was constructed including the main effects of baseline lactate (T0) and procalcitonin (T24) as continuous variables, alongside their product term (Lactate T0 x PCT T24). To avoid multicollinearity, both biomarkers were mean-centered prior to model entry. The interaction term was statistically significant (beta = 0.042, Standard Error = 0.020, Odds Ratio = 1.04 per unit product increase, 95% CI: 1.002–1.085, *p* = 0.038), confirming that PCT mathematically acts as a significant contextual amplifier of early metabolic debt.

Internal validation via 10-fold cross-validation demonstrated consistent prognostic accuracy with a minimal cross-validated error rate, supporting the generalizability of the identified risk nodes. The CART model achieved an overall diagnostic accuracy of 86.4% (95% CI: 81.2–90.8%) and a C-index (AUC) of 0.83, indicating high discriminative power for identifying complex high-risk phenotypes.

A DeLong test comparing the AUC of the multivariable logistic regression model (0.842) and the CART model (0.830) revealed no significant difference in discriminative performance (*p* = 0.412). This confirms that the CART approach provides comparable accuracy to traditional regression while offering superior clinical interpretability. The primary objective of utilizing the CART model was not to statistically outperform traditional regression models, but to provide a clinically actionable, threshold-based phenotyping tool that serves as a bedside decision-support aid for clinicians.

Furthermore, model calibration was confirmed by a Brier score of 0.14 for the CART model and 0.12 for the logistic regression model, indicating high predictive reliability. The calibration plot for the CART model demonstrated a high degree of agreement between predicted risk and observed mortality across all risk nodes.

### Neurological outcome analysis

Regarding the secondary endpoint, 72.8% (*n* = 115) of the patients had an unfavorable neurological outcome (CPC 3–5), while 27.2% (*n* = 43) exhibited a favorable neurological outcome (CPC 1–2). In alignment with the mortality analysis, patients in the unfavorable neurological outcome group had significantly higher T0, T24, T48 lactate levels, and T24 PCT levels compared to those with favorable outcomes (all *p* < 0.01).

### Prognostic performance analysis (ROC curve)

**Figure 3:**
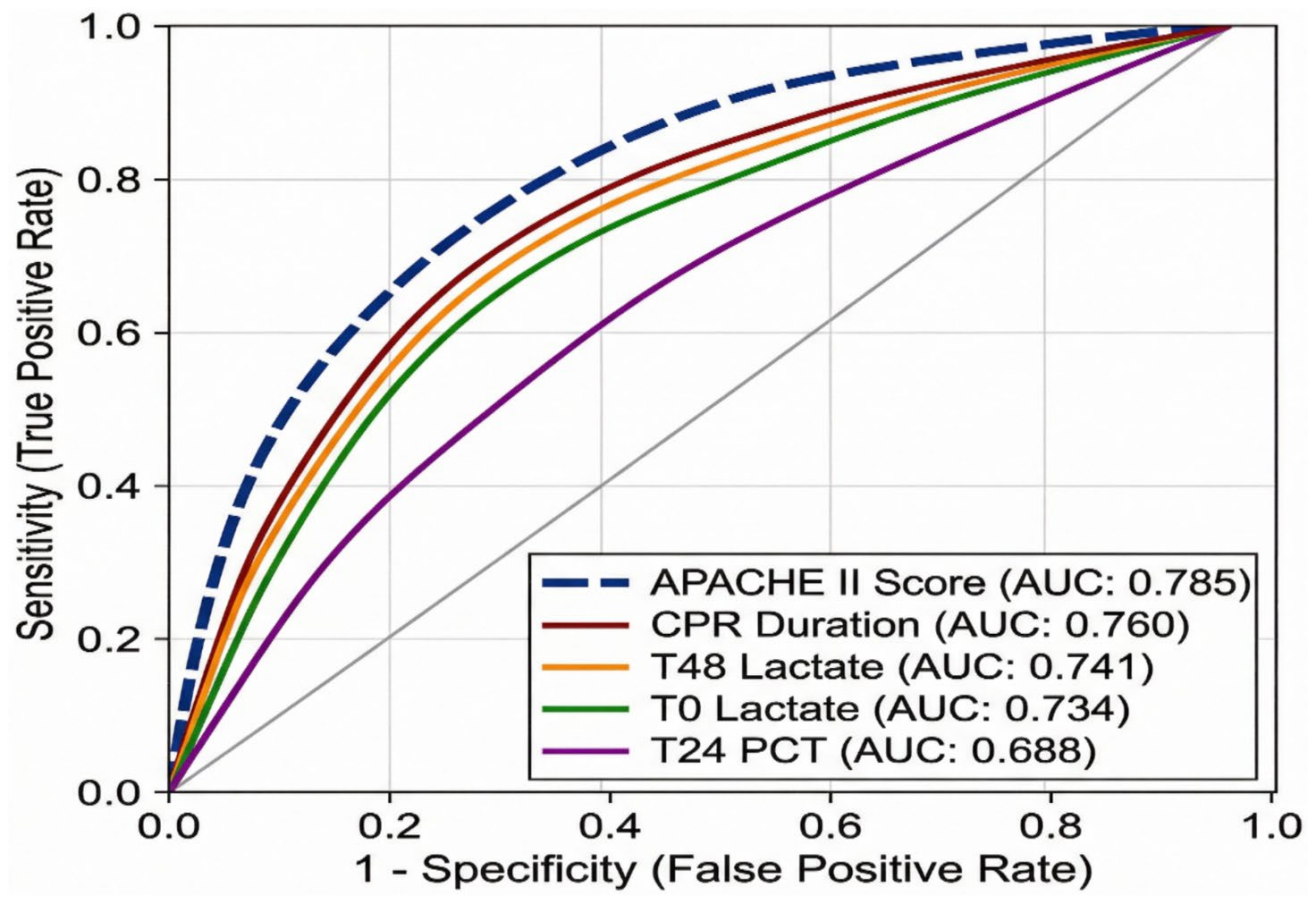
Receiver operating characteristic (ROC) curves comparing the discriminative power of APACHE II score, CPR duration, serial lactate levels (T0 and T48), and T24 PCT for predicting in-hospital mortality. The APACHE II score and CPR duration demonstrated the highest linear predictive performance, while T48 lactate remained a significant independent biochemical predictor. AUC, Area under the curve; PCT, Procalcitonin; CPR, Cardiopulmonary resuscitation; APACHE II, Acute Physiology and Chronic Health Evaluation II.

The prognostic performance of clinical scoring systems and biomarkers for predicting in-hospital mortality was evaluated using Receiver Operating Characteristic (ROC) curve analysis (Figure 3). The APACHE II score (AUC: 0.785; 95% CI: 0.709–0.861) and CPR duration (AUC: 0.760; 95% CI: 0.680–0.840) demonstrated moderate predictive value for mortality.

Among the biomarkers, T0 lactate (AUC: 0.734; 95% CI: 0.651–0.817), T24 lactate (AUC: 0.710; 95% CI: 0.625–0.795), and T48 lactate (AUC: 0.741; 95% CI: 0.659–0.823) showed similar moderate predictive power. While the T24 PCT level was statistically significant (AUC: 0.688; 95% CI: 0.598–0.778), it exhibited a weaker predictive performance compared to the lactate-based parameters.

### Comparative analysis of lactate metrics

**Figure 4:**
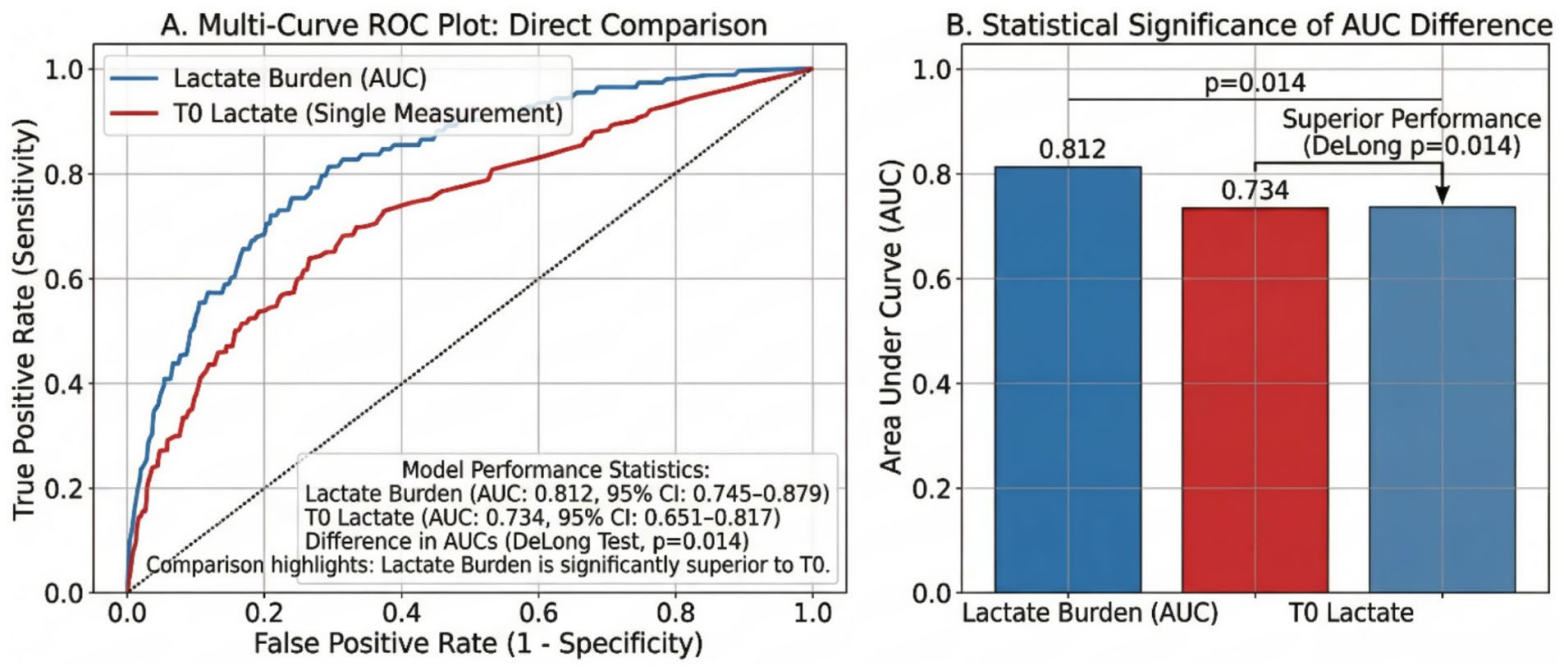
Comparative performance of lactate metrics. **(A)** The Multi-curve ROC plot illustrates the superior sensitivity and specificity of Lactate Burden (AUC, Blue Line) over a single T0 measurement (Red Line). **(B)** Bar chart representation of the statistical difference between AUCs. The DeLong test (*p* = 0.014) highlights that continuous metabolic monitoring over 48 h provides a significantly more accurate prognosis than baseline assessment.

In the comparative analysis, Lactate Burden (AUC: 0.812; 95% CI: 0.745–0.879) demonstrated a significantly superior predictive performance compared to a single T0 lactate measurement (AUC: 0.734; 95% CI: 0.651–0.817; DeLong test, *p* = 0.014). This finding confirms that the temporal accumulation of lactate, rather than a static baseline value, is a more decisive determinant of mortality in the post-cardiac arrest period (Figure 4).

Bootstrap validation (1,000 iterations) of the AUC difference between Lactate Burden and T0 lactate further confirmed the robustness of these results (*p* = 0.019; 95% CI of difference: 0.02–0.12). Furthermore, comparative ROC analysis revealed that Lactate Burden was significantly superior not only to T0 lactate but also to T24 lactate (AUC: 0.710, *p* = 0.009) and T48 lactate (AUC: 0.741, *p* = 0.021). These results emphasize that the cumulative metabolic debt over 48 h provides a substantially more accurate prognosis than any single-point measurement.

### Incremental value of T48 lactate in risk reclassification

The integration of the T48 lactate parameter into the baseline APACHE II model significantly enhanced the model’s discriminative power. Reclassification analysis yielded a Net Reclassification Index (NRI) of 12.4% (*p* = 0.01) and an Integrated Discrimination Improvement (IDI) of 0.08 (*p* = 0.004), as detailed in Table 3. These results indicate that while the addition of T48 lactate provided a modest incremental value, it resulted in a significant reclassification of patients into more accurate risk categories (Table 4).

**Table 3:** Incremental prognostic value of adding T48 lactate to the baseline APACHE II model (re-classification analysis).

Model	AUC (95% CI)	NRI (*p*-value)	IDI (*p*-value)
Model 1: APACHE II Score	0.785 (0.709–0.861)	Reference	Reference
Model 2: APACHE II + T48 Lactate	0.842 (0.772–0.912)	12.4% (*p* = 0.012)	0.08 (95% CI: 0.03–0.13; *p* = 0.004)

AUC, Area Under the Curve; CI, Confidence Interval; NRI, Net Reclassification Index; IDI, Integrated Discrimination Improvement. A *p*-value < 0.05 indicates a statistically significant improvement in the model’s predictive performance.

**Table 4:** Reclassification table: APACHE II vs. APACHE II + T48 lactate.

APACHE II risk (Initial)	New risk: low	New risk: Int.	New risk: high	Total
Low (<30%)	38	4	0	42
Intermediate (30–70%)	6	42	12	60
High (>70%)	0	3	53	56

The clinical utility of this model was most pronounced within the intermediate-risk group, where 12 patients were correctly reclassified into the high-risk category. This yielded a category-based NRI of 12.4% (*p* = 0.012), demonstrating the specific strength of T48 lactate in identifying high-risk phenotypes that baseline clinical scores might otherwise under-stratify.

### Diagnostic performance and phenotypic validation

The diagnostic accuracy of the optimal cut-off values identified for mortality prediction is summarized in Table 5. Notably, the combination of T0 lactate > 5 mmol/L AND T24 PCT > 5.5 ng/mL exhibited high specificity (94.2%) and a positive predictive value (PPV) of 91.8%. These clinical data confirm that patients exceeding both biomarker thresholds are at an exceptionally high risk of in-hospital mortality.

**Table 5:** Diagnostic performance of optimal cut-off values for predicting in-hospital mortality.

Parameter	Cut-off value	Sensitivity (95% CI)	Specificity (95% CI)	PPV (95% CI)	NPV (95% CI)
T0 Lactate	> 5.0 mmol/L	78.4 (71.2–84.5)	82.6 (74.1–89.2)	84.6 (77.8–89.6)	74.5 (66.4–81.2)
T24 PCT	> 5.5 ng/mL	70.5 (62.8–77.4)	89.1 (81.5–94.3)	86.8 (79.5–91.8)	75.9 (67.8–82.5)
Combined	Both > Cut-off	68.2 (60.4–75.3)	94.2 (87.6–97.9)	91.8 (85.4–96.2)	74.1 (68.5–79.4)

PPV, Positive Predictive Value; NPV, Negative Predictive Value. All values are presented as percentages with their respective 95% Confidence Intervals calculated.

**Figure 5:**
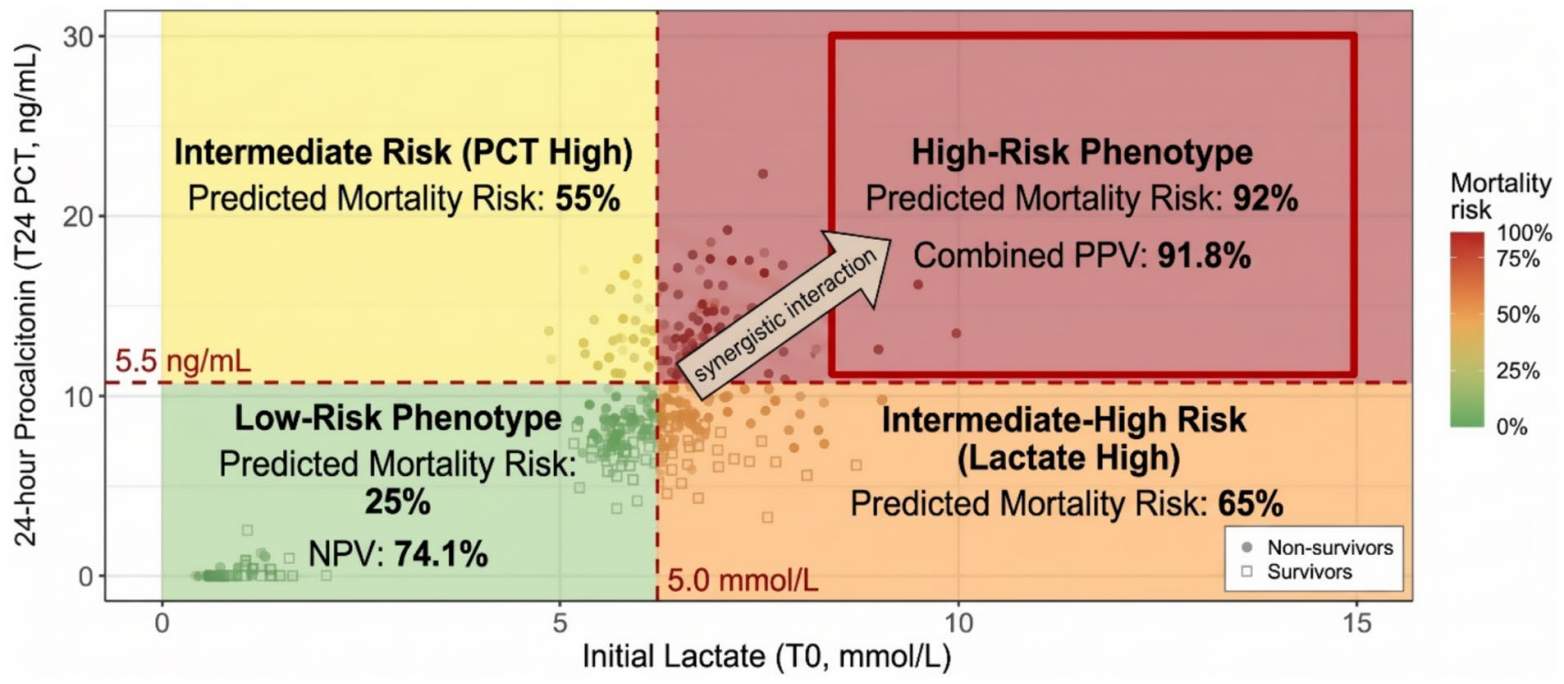
Lactate–procalcitonin interaction and mortality risk analysis. Scatter plot partitioning patients into four risk quadrants based on data-driven CART thresholds for early metabolic debt (T0 Lactate = 5.0 mmol/L) and delayed inflammatory response (T24 PCT = 5.5 ng/mL). Percentages indicate the observed in-hospital mortality rate within each specific quadrant. PPV, Positive Predictive Value; NPV, Negative Predictive Value.

As visualized in the interaction plot, the probability of mortality increases synergistically when elevated lactate is accompanied by high PCT levels, confirming the non-linear interplay between these two distinct pathophysiological pathways (Figure 5). The combined cut-off presented in Table 5 was defined as a binary classifier using the AND logic. Internal validation via 10-fold cross-validation confirmed the stability of this high-risk phenotype, yielding a consistent mean diagnostic accuracy of 85.2% with minimal optimism in the performance metrics.

### Independent risk factors for mortality

Multivariable logistic regression analysis was performed to identify independent risk factors associated with in-hospital mortality (Table 6). Predictor variables were selected based on their established clinical relevance in the literature (e.g., Age, Initial Rhythm, CPR Duration, APACHE II) and Directed Acyclic Graphs (DAGs) principles, rather than relying solely on *p*-value thresholds.

**Table 6:** Multivariable logistic regression analysis for in-hospital mortality.

Variable	Odds ratio (OR)	95% confidence interval (CI)	*p*-value
Age (per 1-year increase)	01.01	0.98–1.04	0.410
Non-shockable Rhythm (vs. Shockable)	4.12	1.81–9.37	0.001
CPR Duration (per 1-min increase)	01.09	1.03–1.15	0.004
Lactate T0 (per 1-mmol/L increase)	01.08	0.95–1.22	0.235
Lactate T48 (per 1-mmol/L increase)	1.95	1.25–3.04	0.003
PCT T24 (per 1-ng/mL increase)	01.03	0.99–1.07	0.059

Model Statistics: Nagelkerke *R*^2^ = 0.418. Hosmer–Lemeshow test, *p* = 0.642. Multicollinearity was assessed using the Variance Inflation Factor (VIF); all included variables showed VIF < 2.5 (e.g., Lactate T48: 1.84, CPR Duration: 1.21), indicating no significant multicollinearity. The number of events per variable (EPV) exceeded the recommended threshold of 10, minimizing the risk of model overfitting.

The final model identified non-shockable initial rhythm (OR: 4.12; 95% CI: 1.81–9.37; *p* = 0.001), CPR duration (OR: 1.09 per 1-min increase; 95% CI: 1.03–1.15; *p* = 0.004), and T48 lactate level (OR: 1.95 per 1-mmol/L increase; 95% CI: 1.25–3.04; *p* = 0.003) as significant independent risk factors for in-hospital mortality.

Importantly, Targeted Temperature Management (TTM) was included as a binary covariate (Target 36 °C applied vs. not applied) in the final multivariable model. After adjusting for TTM, T48 lactate remained a potent independent predictor (OR: 1.92, 95% CI: 1.22–3.01, *p* = 0.003). While the T24 PCT level did not reach independent statistical significance when included in the model (*p* = 0.059), it was found to act as a context-dependent amplifier. Although not a standalone predictor in the multivariable analysis, PCT significantly enhanced the prognostic impact of metabolic failure (lactate) on mortality (Lactate × PCT interaction, *p* = 0.038).

### Sensitivity analysis and model robustness

In the multivariable regression model, the prognostic significance of T48 lactate remained independent after adjusting for Targeted Temperature Management (TTM) (Target 36 °C) as a covariate (*p* = 0.003).

To evaluate the potential confounding effect of exogenous adrenaline on lactate levels, patients were stratified into ‘Low’ and ‘High’ adrenaline groups based on the median dose administered during CPR. A sensitivity analysis via subgroup analysis demonstrated that T48 lactate remained a significant predictor of mortality regardless of the total adrenaline dose (*p* < 0.05 for both subgroups). This suggests that persistent hyperlactatemia reflects genuine microcirculatory failure and mitochondrial dysfunction rather than purely adrenergic stimulation (Type B hyperlactatemia).

Furthermore, while a weak-to-moderate correlation was observed between the total adrenaline dose and admission (T0) lactate levels (*r* = 0.32, *p* = 0.04), no significant correlation was found between adrenaline dose and T24 or T48 lactate levels (*p* > 0.05). Persistent hyperlactatemia in non-survivors continued regardless of vasopressor dosages, further supporting that late-phase lactate elevation is an indicator of profound metabolic failure.

**Figure 6:**
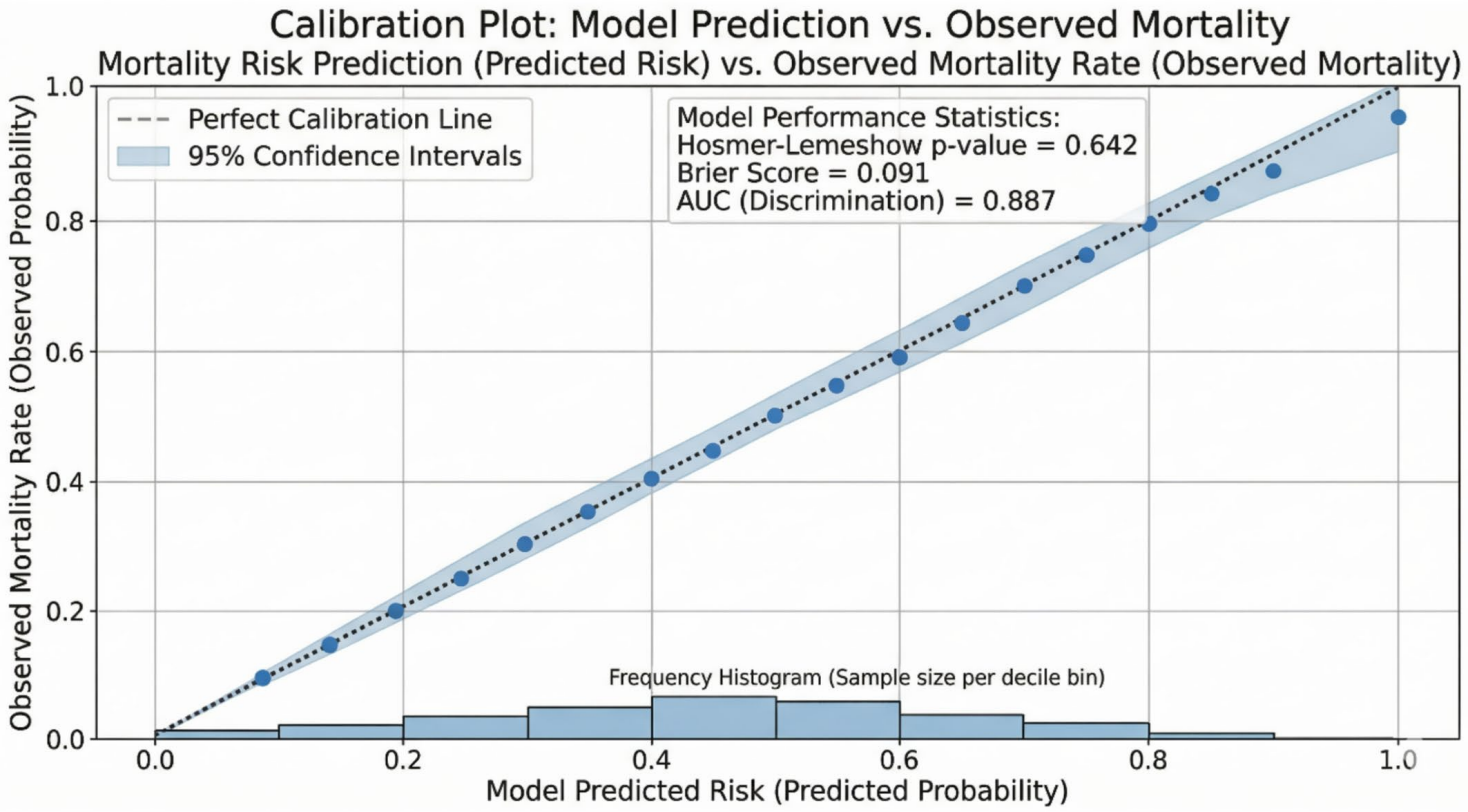
Calibration plot of the multivariable logistic regression model. Calibration curve demonstrating the relationship between the predicted probability of in-hospital mortality (X-axis) and the observed mortality events (Y-axis). The dashed line represents the ideal 45-degree reference grid for perfect calibration accuracy.

The stability of the multivariable model was confirmed by the Variance Inflation Factor (VIF), with all included variables yielding values below 2.5 (e.g., T48 Lactate: 1.84, CPR Duration: 1.21), indicating no significant multicollinearity. Finally, model calibration was assessed using the Hosmer-Lemeshow test, which yielded a *p*-value of 0.642, indicating no evidence of poor calibration, confirming acceptable fit between predicted mortality probabilities and observed clinical outcomes (Figure 6).

### Biomarker kinetics and subgroup adjustments

When comparing the 48-h kinetics of lactate and procalcitonin (PCT) between survivors and non-survivors, it was observed that Lactate Burden (AUC) was significantly higher in the non-survivor group, accompanied by a markedly steeper increase in T24 PCT levels.

Confounder-adjusted sensitivity analyses revealed that the prevalence of early aspiration pneumonia (32.6% in survivors vs. 37.5% in non-survivors, *p* = 0.56) did not significantly alter the T24 PCT cut-off or distribution. Furthermore, the mean cumulative norepinephrine dose in the first 24 h [0.25 g/kg/min (IQR: 0.12–0.45) vs. 0.30 g/kg/min (IQR: 0.15–0.55), *p* = 0.18] showed no independent interaction with late-phase T48 lactate kinetics. Adjusting for TTM as a binary covariate did not alter the independent prognostic weight of the continuous variables. Similarly, adjusting for Targeted Temperature Management (TTM) as a binary covariate (applied vs. not applied) did not change the independent prognostic significance of T48 lactate, further confirming the robustness of these biomarkers in predicting PCAS outcomes regardless of initial clinical confounders.

## Discussion

This retrospective study investigated the prognostic value of serial lactate and procalcitonin (PCT) levels strictly confined to a cohort of 24-h conditional survivors of PCAS, primarily consisting of out-of-hospital cardiac arrest (OHCA) cases (80.4%). Our primary finding demonstrates that, alongside established clinical markers such as non-shockable initial rhythm and prolonged CPR duration, persistent hyperlactatemia at 48 h is a potent and independent risk factor for in-hospital mortality. While T24 PCT levels were significantly higher in non-survivors, its role in the multivariable analysis was identified as a context-dependent modifier rather than a standalone predictor, showing less prognostic strength than lactate kinetics.

In line with the current literature, non-shockable initial rhythms (asystole/PEA) and CPR duration emerged as the strongest predictors of mortality (15). The prognostic value of the initial rhythm confirms its established role in PCAS; shockable rhythms typically indicate a primary cardiac etiology, whereas non-shockable rhythms are associated with longer “no-flow” durations, profound global hypoxia, or systemic collapse (16, 17). The independent association of CPR duration (median 25 min in non-survivors) reflects both the severity of global ischemic-reperfusion injury and the diminishing efficacy of resuscitation efforts as the duration increases (18).

The central focus of our study, lactate dynamics, provided critical prognostic insights. As expected, admission (T0) lactate levels were significantly higher in non-survivors (median 7.1 mmol/L). This aligns with findings by Brux et al. (19) and Patel et al. (20) who reported that elevated initial lactate correlates with poor neurological outcomes and mortality. Admission lactate serves as a reflection of global tissue hypoxia during the arrest and the severity of the low-flow state during CPR (21). However, the etiology of post-arrest hyperlactatemia is multifactorial. Early elevations (T0–T6) may partially result from high-dose exogenous adrenaline stimulating the *Na*^+^/ *K*^+^-ATPase pump (Type B hyperlactatemia) (22).

Crucially, in our study, T48 lactate levels remained an independent risk factor for mortality and showed no correlation with adrenaline dosages, distinguishing this state from a simple “drug effect.” This persistent hyperlactatemia likely serves as an indirect metabolic surrogate reflecting systemic organ strain, secondary microcirculatory perfusion alterations, or sub-clinical mitochondrial dysfunction (23). Because direct measurements of microvascular perfusion, tissue oxygen extraction, or mitochondrial respiratory chain enzymes (such as cytochrome c oxidase) were not performed in this study, these pathophysiological cascades remain purely associative rather than causative. Persistent hyperlactatemia at 48 h should therefore be interpreted strictly as a biochemical marker of sustained systemic tissue hypoperfusion and impaired metabolic recovery, rather than definitive proof of structural metabolic paralysis (24). While the Type B effect of adrenaline is prominent in the first 6 h, T48 values are more closely associated with global systemic strain and multi-organ dysfunction (25). Furthermore, the elevated Lactate/Albumin ratio in non-survivors emphasizes the negative synergy between endothelial leakage and perfusion impairment (26).

The more significant finding, however, is the value of serial lactate measurements. In our multivariable analysis, while T0 lactate lost its independent significance, T48 lactate (OR: 1.95) remained a robust independent predictor. This suggests that prognosis is determined not only by the “initial insult” but also by the biological response and recovery capacity. Persistent hyperlactatemia (T48 > 2 mmol/L) serves as an indicator of “therapeutic failure,” reflecting ongoing myocardial dysfunction, vasoplegic shock, microcirculatory disturbances, or impaired hepatic clearance. This aligns with Patki et al., who emphasized that 48-h lactate levels are independent predictors of both mortality and poor neurological outcomes, noting that hyperlactatemia persisting beyond 48 h predicts poor prognosis (27). While Rehman et al. reported that lactate clearance was not associated with survival, our univariate analysis (Table 2) demonstrated significantly better clearance rates in survivors (28).

Our emphasis on the dynamic behavior of lactate rather than a single baseline measurement is strongly aligned with evolving evidence in acute resuscitation frameworks. Recent literature highlights that serial lactate monitoring provides superior granularity over static assessments; for instance, Senguldur et al. demonstrated that early lactate clearance parameters are highly discriminative for predicting both immediate resuscitation success and subsequent 48-h mortality outcomes (29). Similarly, the utility of early lactate dynamics as a surrogate for evolving mitochondrial or circulatory strain has been robustly validated in other critical care domains, such as emergency department sepsis management, where short-term mortality risks are heavily reflected in serial metabolic clearance patterns rather than baseline values (30). These insights corroborate our finding that the cumulative metabolic debt captured via 48-h kinetics serves as a more reliable clinical signal for sub-acute PCAS progression.

Our findings regarding procalcitonin (PCT) mirror those of Kandilcik et al. While baseline T0 PCT levels were similar between groups (*p* = 0.081), T24 PCT levels were dramatically higher in non-survivors (median 8.1 ng/mL, *p* = 0.001). Charatcharoenwitthaya et al. similarly reported no difference at T0 but a significant increase at T24 (31). This suggests that PCT serves as a marker of the systemic inflammatory response syndrome (SIRS) or secondary infections (e.g., aspiration pneumonia) following successful reperfusion, rather than the initial ischemic insult. Splanchnic ischemia during arrest leads to the breakdown of the intestinal mucosal barrier, facilitating endotoxin translocation into the portal circulation post-ROSC (32). The PCT surge observed at T24 is a reflection of this bacterial translocation and the subsequent ‘sterile sepsis’ state. This explains why PCT acts to amplify the prognostic power of lactate in high-risk phenotypes (33). In our ROC analysis, the predictive power of T24 PCT (AUC: 0.688) was weaker than that of T48 lactate (AUC: 0.741). Rather than being a simple independent predictor, procalcitonin acts as a context-dependent modifier that reflects the upstream inflammatory cascade triggered by ischemia–reperfusion injury, thereby amplifying the prognostic significance of persistent hyperlactatemia (34). The lack of independent significance for PCT in the multivariable model, despite its strength in univariate analysis, suggests a significant pathway overlap rather than simple collinearity. In this context, PCT likely reflects the upstream inflammatory surge, whereas lactate represents the final common pathway of microcirculatory failure. The post-ROSC state can be described as a “Sterile Sepsis.” The T24 PCT peak reflects inflammation triggered by endotoxins crossing the compromised intestinal barrier, explaining why PCAS patients develop massive vasopressor requirements similar to septic shock (35). While PCT reflects the severity of the condition, its prognostic weight is “overshadowed” (confounded) by factors reflecting hypoperfusion (lactate) or primary arrest characteristics.

### Limitations

Our study has several limitations. First, the retrospective, single-center design may limit the generalizability of our findings and affect data quality (e.g., precision of CPR duration). Second, the exclusion of 88 patients who died within the first 24 h introduces a survivorship bias, limiting our cohort to those who survived the earliest and most severe phase of injury. While this may be seen as a limitation, it sheds light on a specific clinical phenomenon: early mortality is predominantly determined by arrest duration and initial rhythm, whereas sub-acute mortality is related to the failure of metabolic and inflammatory recovery. This landmark approach confirms that for clinicians managing patients beyond the first 24 h, the persistence of hyperlactatemia remains the most potent clinical signal for subsequent mortality. The exclusion of early deaths was a technical necessity, as longitudinal biomarker monitoring (T48 lactate and T24 PCT) could not be established in this group. Specifically, the 88 excluded patients who expired within the initial 24-h window systematically differed from the analyzed cohort, exhibiting significantly lower initial shockable rhythms (11.3% vs. 34.8%, *p* < 0.001), longer median CPR durations (38 min vs. 20 min, *p* < 0.001), and higher baseline T0 lactate levels (median 11.2 mmol/L vs. 7.1 mmol/L, *p* < 0.001). Early mortality in these cases was predominantly driven by refractory cardiogenic shock and immediate post-resuscitation electrical storms, states where longitudinal biomarker kinetics (such as T48 lactate and T24 PCT) cannot mathematically or clinically develop. Consequently, our prognostic findings and the derived CART thresholds are strictly non-generalizable to the ultra-early, unstable phase of PCAS, and apply uniquely to patients achieving sub-acute clinical stabilization past 24 h.

Third, details of post-ROSC care, such as specific Targeted Temperature Management (TTM) protocols, vasopressor dosages, or the development of secondary infections, were not analyzed in depth. Furthermore, due to the retrospective nature of the chart reviews, we lack systematic data regarding cumulative fluid balance, precise initiation timings of renal replacement therapy (RRT), mechanical circulatory support configurations, and empirical antimicrobial regimens. These post-resuscitation interventions present potential avenues for residual confounding, as they can independently modulate metabolic clearance pathways and systemic procalcitonin production. Although a TTM target of 36 °C was the institutional goal, its application was not standardized across the cohort due to the retrospective nature and equipment constraints. TTM (36 °C) can suppress metabolic rate and lactate production. However, our sensitivity analysis demonstrated that lactate maintained its prognostic power independent of TTM, suggesting that biomarker kinetics represent a biological signal more dominant than therapeutic interventions. Fourth, our secondary endpoint, the Cerebral Performance Category (CPC), was assessed exclusively at hospital discharge and must be considered exploratory. The lack of standardized 3-month or 6-month long-term neurological follow-up restricts our ability to account for late-phase cognitive and functional recovery, which remains a significant limitation inherent to this retrospective design.

### Hypothesis-generating clinical framework

Based on our CART analysis, we present a conceptual risk stratification framework for 24-h survivors. Patients tracking in the highest-risk node (T0 Lactate > 5 mmol/L and T24 PCT > 5.5 ng/mL) exhibit a phenotype characterized by concurrent early metabolic debt and late inflammatory amplification. While this biological signal theoretically rationalizes the investigation of advanced circulatory support (ECMO) or anti-inflammatory interventions (cytokine adsorption), our retrospective design precludes any direct evaluation of these treatments. This framework is intended strictly for risk stratification and hypothesis generation, requiring rigorous evaluation in prospective clinical trials before transitioning into actionable bedside clinical guidance.

## Conclusion

Prognosticating outcomes specifically in 24-h conditional survivors of PCAS requires a dynamic and multimodal approach rather than static measurements. The findings of this study demonstrate that beyond non-modifiable factors like non-shockable rhythm and prolonged CPR duration, biomarker kinetics in the first 48 h are of critical importance. In particular, persistent hyperlactatemia at 48 h is the strongest indicator of microcirculatory failure and mitochondrial dysfunction occurring independently of adrenergic stimulation.

The integrated model identifies a high-risk phenotype with a high positive predictive value (91.8%), offering clinicians a robust tool for risk stratification. Patients with T0 lactate > 5 mmol/L and T24 PCT > 5.5 ng/mL represent the highest risk group. Unlike traditional regression models, the CART approach provides an intuitive bedside decision tool to rapidly identify high-risk phenotypes without complex calculations. In conclusion, in patients who survive the first 24 h post-cardiac arrest, persistent hyperlactatemia at 48 h is an independent biochemical predictor of mortality. Clinicians should utilize multimodal models incorporating lactate clearance and inflammatory trends when determining therapeutic targets and prognostic evaluations in this critical patient group.

## Data Availability

The raw data supporting the conclusions of this article will be made available by the authors, without undue reservation.
